# Hepatic portal venous gas: A case report and review of literature

**DOI:** 10.4103/0972-5229.56058

**Published:** 2009

**Authors:** Vikas Kesarwani, Dhaval R. Ghelani, Graham Reece

**Affiliations:** Department of Intensive Care Medicine, Blacktown Hospital, Blacktown Road, Blacktown NSW 2148, Australia

**Keywords:** Ischemia, gas, mesentery, portal vein

## Abstract

Hepatic portal venous gas (HPVG) results from mesenteric ischemia and a wide variety of other causes. The primary factors that favour the development of this pathologic entity are intestinal wall alterations, bowel distension, and sepsis. Findings of HPVG during an ultrasound or computed tomography (CT) scan should be carefully evaluated in the context of the clinical picture. In the absence of features of bowel ischemia, the prognosis of patients with HPVG is usually good.

## Introduction

Air in the liver has intrigued the doctors since the middle of the last century when it was first described by Wolf and Evans[1] in infants dying of abdominal catastrophe. Air in the liver can be either in the portal venous system or the hepato-biliary tree and seems to be more common than realised.

We present a case of air in the portal venous system and review the literature regarding its evolution, etio-pathogenesis, investigation modalities, and prognosis.

## Case Report

A 74-year-old Caucasian female was admitted to the hospital for rapid atrial fibrillation and lower limb cellulitis. While being treated with anti-arrhythmic agents and antibiotics, she developed non projectile bilious vomiting and watery diarrhoea. Her surgical history included an appendicectomy 17 years ago and a hysterectomy followed by radiotherapy for endometrial carcinoma seven years ago. Her regular medications were digoxin, warfarin, clopidogrel, and metoprolol.

On clinical examination, she appeared in no acute distress. Her heart rate was 134 per minute in atrial fibrillation, her blood pressure was 124/56 mmHg, her respiratory rate was 25/min, and her temperature was normal. The abdomen was distended with increased bowel sounds and mild tenderness in the right iliac fossa. There was no rebound tenderness, rigidity, or guarding and no discrete mass was palpable. The rest of the systemic examination was unremarkable. Blood investigations revealed deranged renal function with a blood urea of 5.2 mmol/L (2.5-6.1) and creatinine of 186 micromoles/L (50-110). Liver function tests were normal with bilirubin 7 micromoles/L (<21), ALT 26U/L (<33), AST 36U/L (<45), GGT 28U/L (<30), and ALP 116 U/L (30–115). Her white cell count was 27.2×10^9^/L (3.9-11.1) and hemoglobin was 14.1 gm/dL with normal coagulation parameters.

**Figure 1:**
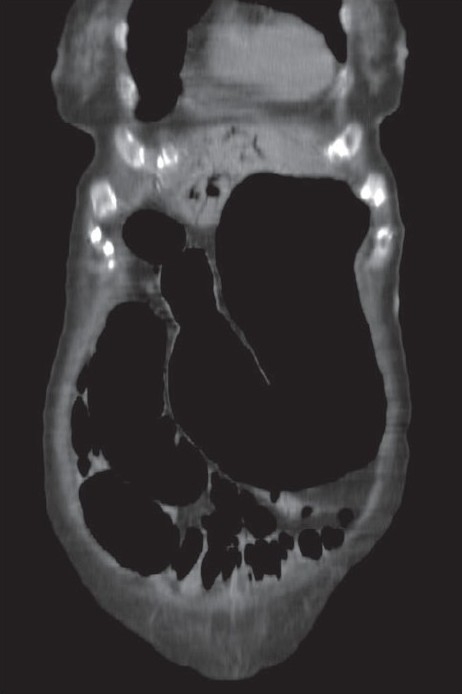
Saggittal section of the abdomen showing severe gastric and intestinal dilatation

**Figure 2:**
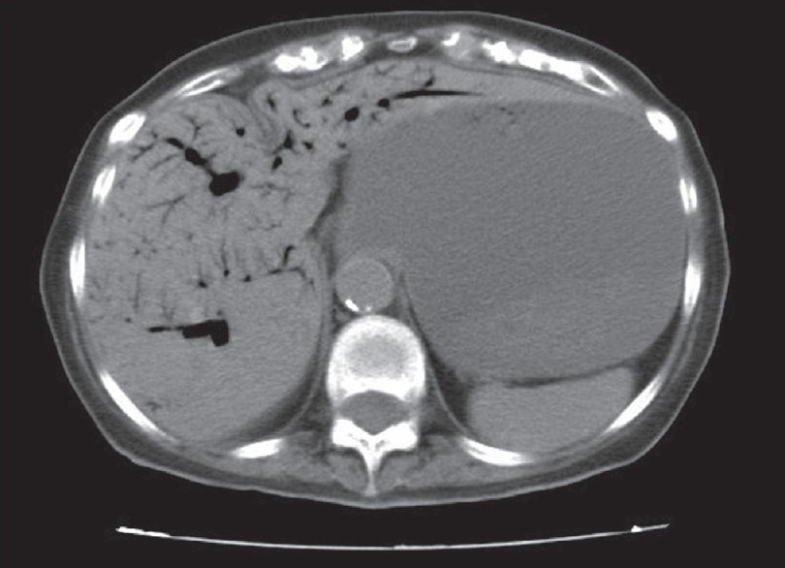
Transverse section at the level of the liver showing tubular areas of decreased attenuation in the periphery of the liver; findings that are consistent with gas in the intrahepatic portal veins

She underwent an oral-contrast enhanced CT scan of the abdomen (intravenous contrast was not administered due to renal impairment), which showed a markedly distended stomach with dilated small bowel loops [Figure 1], but free gas or fluid collection were not noted. The presence of gas in the distended bowel wall could not be ruled out. There were multiple streaks of gas in a branching pattern seen throughout the liver parenchyma [Figure 2]. Her history was revisited but there was no previous hepatobiliary disease or endoscopic retrograde cholangiopancreatography.

In view of the possibility of intestinal obstruction with a low probability of bowel necrosis, the patient underwent an exploratory laparotomy. The operative findings were small bowel obstruction due to fibrotic adhesions with no visible evidence of intestinal ischaemia or necrosis. The abdomen was closed after resection of the adhesions. Her bowel function returned to normal in 3 days and she was discharged from the hospital after 2 weeks.

## Discussion

Air in the liver is not a specific disease entity but merely another diagnostic clue for patients suffering from an acute abdomen. The occurrence of gas in portal veins has been described in literature as pneumoportogram, gas embolisation of the portal vein or portal venous gas. It was first described as hepatic portal venous gas (HPVG) in 1978 by Libman, *et al*.[2]

Until 1968, HPVG was viewed as an inevitable harbinger of death with a mortality rate of over 90%. Libman, *et al*.[2] reported 64 cases of HPVG with an overall mortality rate as high as 75%. In their review of 182 cases of HPVG, Hiroyuki Kinoshita, *et al*.[3] noted that the mortality rate was very high (75%) in 79 cases of HPVG involving bowel necrosis, but in another 81 cases, the mortality rate was less than 30% in those caused by digestive tract dilatation, abscess, and gastric ulcer. The overall mortality rate was 39%. Interestingly, only 46% of the cases underwent an operation and there were no lethal cases of HPVG associated with ulcerative colitis, intraperitoneal tumor, Crohn's disease, cholangitis, pancreatitis, or complications of endoscopic procedures. In another outcome case series[4] of 17 patients with portal venous gas detected by contrast tomography of varied etiology, Faberman and Mayo-smith reported a mortality rate of 29% depending on the underlying etiology. This decline in mortality rate is considered to be the result of early diagnosis as well as recognition of an increasing number of clinically unimportant causes of HPVG by the frequent use of ultrasonography and CT scans.[5]

HPVG is most often confused with pneumobilia viz. gas in the hepato-biliary tree. The pattern of air in HPVG has been described as that extending to within 2 cm of the liver capsule as seen in Figure 2, while the air in the biliary tract remains central around the portal hilum and does not extend to within 2 cm of the liver capsule. The gas in the biliary tree moves towards the portahepatis by the centripetal force of the bile and appears central in the liver, while that in HPVG travels peripherally caused by the centrifugal flow of blood. HPVG is commonly associated with intramural bowel gas called pneumatosis intestinalis, which may be secondary to necrotic bowel. It is characterized by thin, continuous, crescenteric radiolucency with in the distended bowel wall. Although the pathophysiology and mechanism of HPVG are not well understood, the factors associated with it include bowel distension with mucosal damage, sepsis, and gas embolization.[6] It is hypothesised that the gas enters the portal circulation through the veins or lymphatics of the intestinal wall and finally reaches the hepatic veins after passing through the hepatic sinusoids. The various reported causes of HPVG are summarised in Table 1.

**Table 1:** Various reported causes of HPVG

**Inflammatory**
- Ulcerative colitis
- Crohn's disease
- Sigmoid diverticulitis
- Acute appendicitis
**Sepsis/Infection**
- Abdominal tuberculosis
- Necrotizing enterocolitis
- Suppurative cholangitis
- Intra-abdominal abscess
**Iatrogenic causes**
- Post endoscopy
- Gastrostomy
- Barium enema
- Sclerotherapy for gastric varices
- Endocopic Retrograde Cholangiopancreatography
- Endoscopic sphincterotomy
- Gastric dilatation
**Pediatric**
- Necrotizing enterocolitis
- Hirschsprung's disease
- Collagen vascular disease
- Hypertrophic pyloric stenosis
**Others**
- Blunt trauma
- Mesenteric infarction
- Intestinal obstruction
- Gastric ulcer disease
- Paralytic ileus
- Caustic ingestion
- Colchicines toxicity
- Seizure
- After hepatic transplantation
- Idiopathic

Portal venous gas has a high carbon dioxide content,[7] which is expected to exist only briefly in the vascular system before being absorbed or removed by bulk flow, unless the gas production persists. The portal venous gas may also be related to an increase in intra-luminal pressure, which forces intra-luminal gas through a damaged or undamaged bowel wall where it is absorbed into the portal circulation. This scenario has been reported in cases of ileus or gastric dilatation, after blunt abdominal trauma, endoscopy, and barium enema examination.[8]

In our case, we contemplate that the cause of the gas in the portal vein was due to stomach and small bowel dilatation with resultant mucosal damage and pneumatosis intestinalis. And the small bowel obstruction was acute due to adhesions from previous abdominal surgery and radiotherapy.

In the setting of intra-abdominal sepsis as a cause for HPVG, gas-forming organisms violate the mesenteric veins to reach the portal system compared with the more common circumstance of intra-luminal gas passing through damaged mucosa.[9,10] However, an increase in intra-luminal pressure alone might cause mucosal damage and allow gas to enter the portal venous system and not necessarily be associated with intestinal necrosis.[10]

The diagnosis of HPVG is usually made by plain abdominal radiography, CT scan, or ultrasonography. The identification of HPVG on plain films might be subtle with peripheral branching linear radiolucencies in the right upper quadrant. Abdominal plain films fail to show portal air in approximately 80% of the cases. Plain abdominal film may occasionally provide evidence of pneumatosis intestinalis involving the large or small bowel.[11] On gray-scale sonography, air in the portal vein can be seen as flowing echogenic bubbles.[10,12] CT scans and ultrasound are more sensitive in depicting smaller amounts of portal venous gas compared with plain radiographs.[13] Depending on the gas load delivered to the liver, the portal flow rate, and the patient's posture, the antero-superior aspect of the left lobe (representing the most anti-dependent portion of the liver) is the most common site for gas accumulation.[14] The contrast-enhanced CT scan has been reported as a powerful investigatory tool to differentiate HPVG with acute mesenteric ischemia from non ischemic cases.[15] Taourel, *et al.*[5] reported sensitivity and specificity of computed tomography for detection of secondary mesenteric ischemia at 83% and 93%, respectively.

The indication for surgery in porto-mesenteric vein gas is based on the underlying cause of the disease entity rather than HPVG per se. Treatment with broad-spectrum antibiotics is sufficient in most cases, while abdominal sepsis and intestinal necrosis warrant surgical intervention.

## Conclusion

Hepatic Portal Venous Gas is not a specific diagnostic entity but a diagnostic clue in patients presenting with abdominal symptoms and signs. Despite the earlier belief of radiographic detection of gas in the portal venous system as a life-threatening sign, HPVG is not in itself a predictor of mortality, which is determined by the underlying pathology.
